# Global South expatriates, homesickness and adjustment approaches

**DOI:** 10.1186/s40985-020-00122-9

**Published:** 2020-05-24

**Authors:** Dieu Hack-Polay

**Affiliations:** Lincoln International Business School, University of Lincoln, Brayford Pool, Lincoln, LN6 7TS UK

## Abstract

**Purpose:**

The research examines homesickness in organisationally assigned expatriates from developing countries or Global South serving in Western contexts. It investigates the extent to which homesickness has personal and organisational consequences and explores the coping mechanisms used by expatriates.

**Design/methodology/approach:**

This is a qualitative research built on unstructured interviews with organisationally assigned expatriates from the Global South.

**Findings:**

The research found that homesickness has consequences for both expatriates and organisations. These consequences include psycho-social disorder, deterioration of physical health which damagingly affects individual well-being, work outcomes and organisational commitment.

**Practical implications:**

The practical implications centre on the opportunity for policy and strategy formulation by international human resource management (HRM) within organisations to improve the mental health of Global South expatriates, thus seeding the ingredients for better performance and job satisfaction.

**Originality:**

This research makes significant additions to the expatriate literature in exposing the homesickness experiences of expatriates from the Global South in advanced economies. Two main coping frameworks used by expatriates are proposed. These copying frameworks centre on positive practices and negative practices which, in turn, encapsulate five adjustment approaches. The research explains how Global South expatriates use these models in practice.

## Introduction

Adjustment is a critical issue facing expatriates [22]. This can cause social isolation and homesickness, which should draw research attention. There are, however, few human resource management (HRM) publications covering these topics. Further, the literature is largely Western-centric, having more prominently considered the experiences of Western expatriates [14, 28]. The work of Wells and Warren [54] is one of the first attempts to report the experiences of developing country expatriates regarding the psychological anxiety, structural and societal issues they faced in Indonesia. Since then the coverage of developing world expatriate experience has been patchy.

It is thought that homesickness is key issue affecting expatriates and causing performance issues in international organisations. Homesickness, an experienced condition of distress, affects people who are away from their usual habitation, navigating unfamiliar sociocultural and physical environments [4, 50]. Thurber and Walton [47] perceive homesickness as an important element of stress that can engender depression and weakening of one’s immune system. This perception is widely supported. Fisher [15] claims that there is medical evidence reinforces the view that homesickness impacts health. Research advocates that homesickness is prevalent in migrants [16, 29, 35, 47]; but this has not been replicated in expatriate research. The relevance of this is compounded by continuous evidence about high failure rate amongst expatriates, and the scarcity of research concerning Global South Expatriates in particular [20]. Despite the criticality of the issue, little in the HRM literature addresses homesickness, particularly for the growing number of expatriates from the developing world or Global South who are now deployed by international organisations to advanced economies. Global South expatriates provide an importance source of market intelligence for companies expanding into emerging economies and, as such, the understanding of how to ensure their success in their Western assignment could be key to competitive advantage. This research, which is situated in the international HRM context, articulates the following research questions: (R1) To what extent does homesickness in Global South expatriates have personal, emotional and performance implications for expatriates? (R2) To what extent pre-departure training and preparation can help deal with homesickness? (R3) What adjustment mechanisms or responses are deployed by Global South expatriates?

The article first discusses Fisher’s [15] homesickness model as the main research framework in view to apply to the context of expatriate training and adjustment. The distinctive significance of the research lies in the depiction of the expatriate experience of professionals from different emerging countries and the development of models of coping approaches.

### Literature review and theoretical framework

#### Theoretical framework

This research examines homesickness in developing expatriates in the light of Fisher’s [15] homesickness model. Medical and psychological research suggests that homesickness presents behavioural, cognitive and physical symptoms [15]. Physical symptoms include stomach discomforts, lack of sleep, eating disorder, headaches and recurrent exhaustion. Cognitive symptoms comprise thoughts fixated on the homeland and sometimes simultaneously undesirable thoughts regarding the host environments, idealising home and being distracted [15]. For Van Tilburg et al. [50], development of apathy and listlessness, deficient initiative-taking and lack of interest in the host context are parts of these symptomatic behaviours. Fisher [15] and Lin [29] observed multifaceted associations between sociocultural navigation and adjustment from a psychological standpoint, although the business consequences of such psycho-social and physiological anomalies encapsulate lack of motivation and absence of a collective performance spirit [10].

Fisher et al. [16] see homesickness as an episodic condition. They recognise that in severe cases, the period during which the expatriate experiences homesickness could be protracted. Owing to its periodicity, the prevalence of homesickness can be hard to assess as a ‘disease’, even by researchers who adopt the psycho-medical approach. Sufferers experience the condition particularly early into the expatriate assignment [50]. In a major study, Fisher et al. [16] found that only 18% of cases were brought to light amongst a group of pupils at a boarding school. Conversely, more profound examination of that educational setting found 60 to 70% homesickness prevalence rate. The limited expatriate homesickness literature signals that many incidences of homesickness are unreported [5].

Fisher’s [15] formulated homesickness prototypes have characteristics similar to the psycho-social framework developed by Lin [29] based on his study of forced migrants. The research examines the characteristics of these models to establish what they can offer to our understanding of the problem of expatriate homesickness. Fisher’s framework has five components detailing how people away from home are affected by homesickness. The loss and attachment model establishes that separating people from their natural sociocultural connections is a loss that metamorphoses into angst, unhappiness and resentment [41]. Apathy and helplessness develop as a result of the persistence of these psychological states [4, 34, 50]. These latter characteristics could lead to dependency and depression.

Interruption and discontinuity is the second component of Fisher’s framework. It suggests that disruption in people’s routines is a major stressor, resulting in the emergence of undesirable emotions, e.g. fear, anxiety and distress [46, 48]. Individuals become helpless since the bases of the practice would have been interrupted. They can effectively be remedied only through essential behavioural or cultural changes. Within the reduced personal control aspect, expatriates lack control in their work and social context. Individuals may lack strategies for coping with the fresh sociocultural, psychological and technological milieu that they perceive to be hostile. Burt [7] contends that feeling homesick can largely be associated with the diminished control that one experiences. Expatriates are forced to espouse roles that would facilitate their connection with the new place, and this is the perspective supported in the change and transition model of Fisher’s homesickness framework. This transitioning period has a high degree of stress [48]. The conflict aspect suggests the expatriates can ‘agree’ to the terms of life in the new environment, i.e. make adjustments to their own assumptions, behaviour and practices but they can resist accommodating irrevocable and uncontainable environmental changes. The expatriate contemplates returning early. Individuals can traverse one, two or all steps of Fisher’s model depending on several factors as earlier argued [4, 15].

Fisher’s [15] model offers some interesting insights into the construction of homesickness and its manifestations. However, it is not without flaws. Numerous authors in the field contest the emphasis on linearity in the sequence of symptoms [12, 45, 51]. These authors reject the linear perspective in homesickness and rather advocate the acknowledgement of complexities in the condition.

#### Expatriates, homesickness and adjustment

The supportiveness of the expatriates’ social and organisational context can determine how the newcomers experience difficulties such as homesickness [13, 34]. Most studies of homesickness using psychological and medical foundations did not involve as much expatriates as migrants generally. There may be significant differences between migrants who can be homesick but nevertheless are committed to their new country; expatriates who know they will go home when their assignment ends can be at greater risk of homesickness; thus, it is not always possible to read across from one set of literature to another. As Madziva [30] argues, for migrants who have fled traumatic events or harsh economic realities back home, the host country represents a safe haven that allows them to start afresh, devoting psychological energies to rebuilding life. Migrants therefore have the impetus of safety in the new country as a helping factor in dealing with homesickness, in contrast with organisationally assigned expatriates.

The international HRM literature stresses that expatriates can face the dilemma leading to questions about whether to follow an international career at home as home internationalists—home employees active in cross-border virtual teams [31]—or continue an international career abroad [14, 41], regarded as a key objective for well-versed global managers. Expatriates are also anxious about their position and adjustment issues on returning home [26]. The dilemma amongst expatriates is exemplified in recent articles [41], highlighting the intense stress caused by international experience.

Acculturative stress is often mentioned in articles on expatriate adjustment to new cultures. Biasi et al. [4] and Dongfeng [11] associate acculturative stress with the effect of displacement and the novelty of surrounding environments. Though this assertion touches on some critical issues linked to displacement, it does not specifically address homesickness which has different manifestations. Acculturative stress emanates from the challenges that expatriates face in adapting to different cultures but this does not necessarily evoke the sense of missing home which, if recurring, aggravates acculturative stress and develops as compulsive excessive disorder or an illness. Zhu et al. [56] recommend an elaborate degree of risk assessment.

Patteson’s [37] fiction Oscar Hijuelos: ‘Eternal Homesickness’ and the Music of Memory has the central imaginary character evoke home as the ‘original’ island, the ‘island at the centre’ being a place ‘impossible to reach’ (p.38). This leads expatriates to idealise home, which diverts them from organisational performance and local adjustment efforts [4]. As Tuan [49] argues, home is both place and space of familiarity, which are inseparable from identity. This renders dislocation difficult to accustom to as identities and cultural routines embody self but also collective identity that operates as a social shield for the individual [24]. Gil et al. [18] discovered substantial variations in acculturative stress suffered by American adolescents of Mexican origin but born abroad and those born in the USA of bicultural parents. This suggests that feelings about home are critical parameters when investigating acculturative stress and social and work performance in new environments [17, 56]. The authors advocate the ‘need to advance from simple linear explanations to multifactorial stress models that will increase our understanding of the acculturative process’ ([18]:41). In fact, Beaverstock [2] argues that there is a concentration of multinational headquarters in a few cities, generally in the West. This means that increasing numbers of expatriates will come from the Global South, suggesting reverse cultural issues that companies’ HRM needs to manage, through novel paradigms.

Pre-departure cross-cultural training (CCT) as a remedy for expatriate stress has been found to have limited effect [38]. There have been some questions about the effectiveness of cross-cultural training for expatriates [25]. However, recent studies increasingly support the view that there is a positive correlation between CCT and expatriate adjustment and performance. For instance, Caligiuri et al. [8] argue that pre-departure CCT enables expatriates to anticipate, evaluate and later successfully deal with the effects of relocation, e.g. culture shock. Nunes et al. [36] similarly emphasise that CCT engenders cultural intelligence and facilitates cross-cultural adaptation, leading to greater expatriate performance. However, despite significant research acknowledging the importance of pre-departure CCT, current practices in many organisations are inadequate [5, 42, 43]. Substantial rethinking of CCT is required to render it responsive to expatriate needs [27]. These studies reinforce our assumptions that underlying expatriate adjustment issues, including homesickness, still lack critical attention.

## Methodology

The research used unstructured interviews to obtain data from twenty participants (fourteen men and six women) from Asia, Middle East and Sub-Saharan Africa, working in various multinationals and international development agencies in London, UK. Despite the limitations of this form of interviews (e.g. volume of data, potential different questions for participants), the unstructured interviews were deemed important for this research to gather in-depth information from the participants. As there is limited research on expatriate experiences of executives from the Global South, it was important to gather sufficiently detailed information as possible from the participants to develop meaningful analyses. Unstructured interviews also allowed the researcher to focus on the participants’ view of the world [21].

The participants were aged between 31 and 54. The base selection criterion was to have been posted in Britain for up to 3 years. Measurements of homesickness are not conclusive in terms of how long the phenomenon lasts, with some studies advocating decline in homesickness within 2 weeks, while others recorded noticeable decline in homesickness only after 7 weeks and in some cases just over 2 years after relocation [45]. The researcher believes beyond 3 years the expatriates would have passed the period of acute homesickness and develop adjusted sufficiently to the new environment. This assumption is embedded in Ying’s [55] study which found a linear decline in homesickness over the first 2 years following relocation. Table 1 depicts the backgrounds of the participants.

**Table 1:** Participant characteristics

Participants	Age	Home country	Company’s sector
Ibrahim	42	Nigeria	Oil & Gas
Sherriff	38	Gambia	Banking
Cheick	35	Senegal	Banking
Abdu	40	Senegal	Tourism
Zak	40	Ghana	Banking
Mariam	39	Malaysia	Banking
Musa	52	Egypt	Tourism
Bobby	46	Mauritius	Health
Aisha	38	Lebanon	International Development
Yacub	54	Saudi Arabia	Oil & Gas
Nadia	37	Nigeria	Insurance
Seba	36	Ivory Coast	Agricultural Products
Aye	45	Nigeria	Oil & Gas
Fanta	31	Algeria	Health
Ali	44	Dubai	Oil & Gas
George	48	Ghana	Banking
Martha	41	Mauritius	Health
Idriss	50	Dubai	Oil & Gas
Esther	36	Ivory Coast	Agricultural Products
John	53	Ghana	Agricultural Products

Snowball sampling was used to contact the participants; each expatriate contacted led the researcher to other contacts. Snowball sampling has limitations, e.g. issues with reliability and bias. The researcher attempted to make the most of the initial contacts obtained which eased the search for suitable participants through snowballing. The use of snowball sampling was also commanded by the limited numbers of developing world expatriates who operate in multinationals in Western cities [28]. The final number of participants was reached through the process of saturation. In total, twenty interviews were deemed adequate as the interviewer reached saturation. In fact, five further interviews were conducted after the first twenty, but these yielded little completely new data. Data saturation can be problematic in qualitative studies as it can be easily assumed that no new information ca be obtained [39, 40]. However, in the context of this study, given the limited number of known Global South expatriates, the researcher was confident about the level of saturation for this exploratory study.

The unstructured interviews explored various topics that are deemed to create or exacerbate homesickness, e.g. relocation motivation, preparations before the assignment and early experience in new environments, settlement and family issues and whether they missed the homeland [13]. The interviews started with a single opening invitation to the participants: ‘Could you tell me about your experience of homesickness when you came for this expatriate assignment?’. In the course of the interviews, the participants were occasionally prompted to ensure that they provided relevant information and did not significantly divert from the main research theme. Such probing questions included: ‘How did homesickness affect you? How did manage this condition? Could you think of a period when it started and when it peaked? Did the organisation help? What could the organisation have done to help?’. The probing questions were central to obtain valuable data that help construct the models of copying strategies. The interviews took place in convenient locations near the participants’ workplace in London to minimise work disruptions and lasted on average 1.5 h. The protocol for data analysis was through a process of review, open coding and categorisation to ascertain the meaning of the participants’ narratives. The first stage of the analysis involved the data being coded into general themes by assigning a word or phrase to each category. The second stage involved axial coding where the data was put back together in new ways by making connections between themes. The researcher effected the analysis case by case in order to identify similarities and differences in the expatriates’ experiences. The coding structure developed captured the distribution of discourses of the participants and aspects of perspectivisation [19]. The main aspect of the expatriates’ discourses that frequently appeared was identified and the emerging patterns were organised. Finally, the research evaluated the emotions expressed by the expatriates to identify the expressed positive and negative strategies in coping with homesickness. The participants’ experiences were analysed in stages with participants’ further support in interpreting some assumptions and statements they made about their adjustment approaches, ensuring at the same time a first level of triangulation of the data. Table 2 shows the coding structure.

**Table 2:** Coding scheme

Variables	First level codes	Sub-codes
**Symptoms of homesickness**	Organisational support	▪ *Pre-departure training* ▪ *In-assignment training* ▪ *In assignment counselling* ▪ *Financial support for home visit*
	Family support	▪ *Phone call from home* ▪ *Social media contacts* ▪ *Spouse reassurance* ▪ *Children’s happiness*
	Community effect	▪ *Community embracing expatriate* ▪ *Existing colleagues making welcome* ▪ *Language barriers* ▪ *Cultural differences*
	Personality effect	▪ *Usually anxious* ▪ *Insecurity* ▪ *Introversion/extroversion*
**Coping strategies**	Organisation	▪ *Creativity* ▪ *Self-involvement in novel work duties* ▪ *Late working*
	Family	▪ *Time with family* ▪ *Regular home visit* ▪ *Visit from family and friends*
	Individual	▪ Outgoing/extroversion ▪ Active communication ▪ Environmental discovery ▪ Reliance on medicine and substances ▪ Introversion

Triangulation was further achieved through participant validation. With the sample of participants being relatively manageable, the author was able to ask each participant 6 weeks after the interviews to check the accuracy of the transcripts and the draft analysis made. Additionally, the author also sought to contrast the findings with the key literature deployed to support the study.

Ethical guidance in relation to consent and data protection was ensured. The participants were contacted through via a snowball process but each individual was presented with the schedule of interview themes and was asked to provide consent based on the information provided. Participants were assured that the data will remain anonymous and that only the research had their details for further contact purposes in view to seek their validation of the transcripts. The researcher has substituted their names with a preferred pseudonym the participants were asked to provide. The researcher has omitted the name of their organisations in order to further ensure anonymity. The participants were also assured confidentiality. An ethics application was submitted to and approved by the University of Lincoln (UK) Ethics Committee.

## Findings

This section analyses the situations that the participants faced, the coping strategies they developed and explanations for their choices. All the participating expatriates confirmed traversing periods of homesickness at certain stages of their assignment. The stage at which the homesickness condition surfaced first varied; this was as early as at the point of departure for some expatriates. However, most respondents became actually homesick after arriving in the host country. Abdu felt homesick before departure:

Two weeks before leaving, I started to have strange feelings about coming to London. My extended family were happy to meet so often for most religious and social events. I thought about how much I’ll miss that (Abdu).

Fourteen participants acknowledged symptoms in the few days preceding departure in the way described by Abdu; however, generally the strongest symptoms of homesickness were more visible 3 months afterwards. Two other expatriates concurred about feeling the deepest sense of homesickness after a year. The expatriates displayed homesickness symptoms notwithstanding moderating elements, e.g. previous expatriate experience, accompanying family members and knowledge of host country. This finding shows the extent of the problem in international assignments.

### Impact of culture distance

There were multiple hitches associated with the experience of our participant expatriates from the Global South. This testifies to the cultural distance (the difference in cultural values between two countries) [3] amongst North and South despite increasing international business and the meeting of cultures. The more distant the cultures are, the more painful the experience of adjustment becomes, heightening homesickness. Ibrahim explained:

To understand British culture was not easy. For example, how to approach people to have a chat; what can offend people isn’t easy to detect. Thus, to avoid conflict, you stay in your corner.

This feeling was widely shared. Nine expatriates voiced frustrations and described the segregated culture in the office. Despite many participants having different perspectives departing from these opinions, the encounters of the aforementioned expatriates confirm the integration difficulties which can prompt feelings of missing the homeland. Such an evaluation is corroborated as all the expatriates reported issues intrinsically linked to both societal and organisational culture which caused an unhomely feeling. Zak concurred:

The office and the physical landscape are reminders that you aren’t at home. The only way you can forget a bit is to have people around that can be friends, not just office mates. But that doesn’t always happen.

### Anxiety

Anxiety was exemplified by the pressure to close cultural fissures. However, anxiety was moderately present elsewhere, e.g. work context (headquarters’ technology, structure, etc.) and in the wider societal fabric (legal frameworks, pace of life, safety, availability of ethnic commodities). Fearing the new environment was acutely felt amongst women expatriates. Mariam explained:

It’s intimidating to go out alone with a headscarf, when there aren’t many women there [in South London] dressed like that. You feel a foreigner and scared of people looking at you, but sometimes shouting or attacking you.

This was presented as a gender issue but could also be a religious or ‘visibly different’ issue. Since there were only six women in the sample, further research is needed.

The expatriates were from urban centres but none like London and the novelty of things, socio-economic structures, the built environment, food and transportation, etc. were reported as overwhelming. Half of the expatriates feared about the organisational and legal appropriateness of their actions that might cause offence to others and harm themselves. Ibrahim explained:

We heard so many things about attacks in London that as a new expatriate I felt scared of going out. I’ve a different attitude now after 3 years. I go out and socialise. But in the first year I was confined to my apartment; I felt withdrawn and depressed.

Having traversed significant psycho-social interruptions associated with risking career moves and separation, eleven explained that serious anxiety was already taking shape prior to departing the home country. Some expatriates reported irregular pace of the heart and stomach-ache. This stage of homesickness was a foremost phase in the expatriates’ assignment as the participants who were less anxious but with higher levels of resilience showed a higher probability to integrate successfully. Reduction in the level of anxiety soothed the undesirable feeling which brought to light the adverse effects of cultural distance and unfamiliar realities which, in turn, are perceived as inexorably foreign with the potential to be detrimental to the expatriate.

### Flaws of pre-departure preparation

Over half of the expatriates’ evidenced issues that increased their sense of missing home, e.g. new responsibilities, novelty of the work and social contexts and multiple issues they have to coordinate, etc. Evidently, they could always return: all they had to do was resign and go home. Obviously, the expatriates missed home but decided to stay because leaving early will signify a personal failure and possibly damage their future career, as reported:

The reason why I hang on and didn’t leave the assignment was that I’d feel a sense of failure and letting down my employer. That could affect whether they give me another posting in the future or promote me at home (Musa).

On whether pre-departure training actually achieved its intended aim of smoothening the transition between home and away contexts, there were mixed expatriate reactions as evidenced in the participant narratives below. Ten participants had some limited pre-departure preparation. The expatriates who received training, except one with significant prior expatriate experience, stressed the overpowering sense of interrupted competence in their assignment. A female expatriate argued:

Pre-departure training was ok, but it didn’t really stress sufficiently what was involved in the actual role. If we’d been involved in meaningful overlaps, that’d provide hand-on experience and a steady introduction to the new workplace and cultural environment (Mariam).

Mariam’s perception of pre-departure training was shared by two other females, Nadia and Aisha. Mental pictures of the homeland recurred because of such issues; this became more pronounced due to support shortages from subsidiary company and headquarters expressed by six of the expatriates. This negatively impacted on their performance. While Mariam and some few other expatriates see some merits in the pre-departure preparation, four expatriates did not find it helpful and this is exemplified in Bobby’s words:

The local reality’s totally different from what HR covered in pre-departure sessions. They said it was easy to source various foods and leisure facilities here. This isn’t true. We stay home at the weekend because the cinema and theatre don’t always show things that interest us. The shops selling African food are mainly in South and North London.

### Homesickness and unsupported grieving

Grief and loss became part of the experience of expatriation for the participants. As the expatriates became lonely, the absence of relatives and friends was felt more deeply. One would reason that in the technological age with Skype, Facetime, WhatsApp, Facebook, etc., new expatriates will overcome loneliness more rapidly. Even where internet facilities were adequate and these technologies available, the expatriates did not make extensive use of them, suggesting that they miss the warmth of face-to-face social interactions with families and friends. The grief was for the ‘lost’ homeland as a physical, sociocultural and linguistic space which symbolised a sense of belonging. Aisha explained:

I often shook my head and said to myself ‘why did I bring my husband to this place where he’s unhappy’. I often isolated myself in a room and cried. I was generally restlessness and miserable.

All the expatriates acknowledged going through a similar process, despite their initial joy about the opportunity to further their professional experience. Yacub argued:

We grieved for the lost homeland, the lost paradise. Being so far, culturally, socially and psychologically isolated, you can only see home as a paradise. In the face of this, careers seem to matter less, which means less job focus.

In the later stages of the assignment, with time and the building of new networks, the expatriates attempted to settle in their professional lives.

### Symptomatic manifestations of homesickness and concerns

The expatriates experienced homesickness in many ways. This included physiological and psychological states that were pathological. These manifestations varied but participants stated significant similarities.

The expatriates observed persistent mood changes and many reported unsettling feelings in the early days of the assignment, which they attributed to dislocation and anxiety. Such fear caused ten expatriates to observe a decrease in their excitement about the role, feeling unhappy and withdrawn. Changes in facial expressions, evasion of personal space, gnashing of the teeth and sometimes becoming aggressive exemplified the deteriorating state of mind in ten expatriates. There was self-realisation of these behavioural conditions but the expatriates blamed others for their ill-psychological health. Yacub’s experience translates the feeling of many of the participants:

Some expatriates were inappropriately rude when they phoned managers and HR for help in the headquarters. I sobbed in the office openly and sometimes quietly at home.

This phase typifies a situation of diminished control because the expatriates experience helplessness in various contexts. The lack of interest was characterised by the respondents not responding to the normal demands of the body and refusing food. Some participants like Cheikh and three female expatriates reported lack of food intake. In Nadia’s words:

I lost weight, experienced frequent mood change. For weeks after arriving, my diet became imbalanced and for some days I didn’t eat.

Medically, some expatriates noted weakness in their joints and had constant headaches. Others fell ill-health physically but were unable to explicitly describe their condition. Some expatriates declined social interactions and confined themselves into isolation. Abdu said:

Loneliness connected me to the past. Past memories kept me going knowing that I’ll one day return to live these realities.

All participants articulated the feeling of being overwhelmed. Organisational structures, work systems, technology, team dynamics, etc. appeared very different from those in place in the homeland. These, combined with the psychological effects of separation, became an uneasy situation. There were performance issues associated with this phase, particularly early in the assignment. Reports of soldiering meant lower productivity at personal and organisational levels. Seba admitted:

Managers were concerned about the many errors I made in my work, causing backlogs and numerous complaints from customers.

To help Seba through the transition into the new position, HR managers provided counselling and support. The lack of focus affected eight other expatriates.

The data revealed that psychological and social disruption in the expatriates was the result of homesickness and lack of preparation and in-assignment support. Homesickness also affected the participants’ physical health. The issue was graver for expatriates who received limited pre-departure CCT. What were their approaches for coping with homesickness? This section identifies some moderating elements that helped manage the homesickness condition.

### Coping strategies

Some participants got more involved in the local communities and social events within the company to avoid social isolation which triggers and exacerbates homesickness. Bobby, who was an accompanied expatriate, explained his fight against homesickness:

I took my wife and kids to several local cultural events which were good to relax and know the locals. We went to parks and my children played with locals. Frequently, we got a childminder for the kids so we could attend social events.

Other expatriates took cultural awareness classes which they claimed helped them meet locals and become less isolated and sad. Some expatriates kept in contacts with their new colleagues in the foreign assignment and at home via emails, traditional letters, telephone and Skype. A female expatriate explained how this approach helped cope with expatriate life in the West:

In Indonesia, the social and office atmospheres were enjoyable because the culture was similar to my country. However, in London, I feel lonely because my boyfriend wasn’t granted a visa to accompany me. I write several letters to him weekly (Aye).

Personal efforts led a number of expatriates to be ‘hooked up’ on host television programmes to learn British culture. For Nadia:

The daily BBC TV series Eastenders became a ‘religious ritual’ for me. I’ll watch it daily. When I couldn’t make it, I’d set up automatic recording to view later (Nadia).

While education and personal efforts had a positive impact on the expatriate, other coping strategies were less constructive. For example, some expatriates became addicted to drugs to help them relax and deal with the pain of isolation and acculturative stress. Fanta reflected on her experience:

When you take medication, at first it’s fine. Drowsiness leads to sleep. However, medication doesn’t clear the thoughts about home when you wake up. Sometimes, you suffer even more from homesickness.

Some expatriates saw leaving their companies to join rivals as an important coping mechanism. Ali contended:

You find other companies whose support package is more attractive and supportive. I know a company that uses community support to help new expatriates. They invite community leaders to come welcome the new expats. I went to an event with MaltCompany (name substituted) which I later applied to.

Other participants attempted to leave them companies, though unsuccessful; but the intension remained firm. George explained:

I thought looking for new opportunities will provide me fresh challenges and stop me thinking that I made a mistake to leave home. It isn’t about more money or better position but change that could heal my feeling homesick.

The next section discusses the main perspectives of the participants and constructs a model of expatriates’ strategies for coping with homesickness during their assignment.

## Discussion

The findings demonstrate the severity of homesickness and its disruptive effect on expatriate mental health, performance and commitment to the organisation. The fact that it is under-explored within HRM and expatriation studies is a disturbing literature omission. Evidence [10, 35, 47] indicates that homesickness has profound negative impacts. According to psychologists, it is an illness that should be seriously researched to find remedies [15, 50]. The respondents reported not having experienced symptoms like these before and linked them to stress. As they did not report pre-existing conditions, their views were congruent with the literature which characterised the symptoms of homesickness. This study shows that expatriates suffering from homesickness display negative attributes, e.g. irritability, sadness, uncooperativeness and lack of initiative (Research Question 1). The frustrations about the incapacity to melt into the new social context increased homesickness. Those expatriates who could speak a second language or a plurality of languages had an advantage in building initial multiple relationships with people from a variety of backgrounds and nationalities [53].

Establishing the additional factors that intensify homesickness in Global South expatriates constitutes the first step in improving individual well-being and engendering a better return on investment from expatriate assignments. Van Tilburg et al. [50] argued that interventions are limited although Fisher’s [15] ‘stress management’ framework found a number of realistic interventions. Zhu et al. [56] see the necessity to address acculturative stress. Thurber and Walton [47] are optimistic and present medical and social interventions which should draw on family support and employers’ roles. As the impact of culture shock may be unavoidable [47], the homesickness coping model proposed in this study is an initial step to address the psycho-social and performance-related trauma faced by Global South expatriates. Coping strategies are summarised in Tables 3 and 4.

**Table 3:** Expatriate homesickness coping approaches (positive practices)

Action	Expectation
**Approach 1: particularities of the social approach**	
Use of social networks Integration with community or expatriate organisations Increased contacts with friends & family at home Use of the nuclear family Cultural exploration and discovery	Psychological balance Ability to rapidly make sense of host realities ‘Kill off’ the sense of missing home Develop new meaningful routines
**Approach 2: particularities of the Education approach**	
Coordinated assistance by employer Pre-departure training, preparation Returnee testimonies and input Overlap Education attendance in host country	Reduced sense of dislocation Preparedness of expatriate or migrant worker Formulation of personal plan prior to departure
**Approach 3: Particularities of the Personal effort**	
Drive to maintain contact at home Willingness to build contacts with host country nationals Self-directed cultural learning Use of previous expatriate experience Social tourism	Cultural immersion Sense of self-worth Development of temporary social networks Cultural exploration and discovery

**Table 4:** Expatriate homesickness coping approaches (negative practices)

Actions	Expectation
**Approach 4: particularities of medicalisation**	
Consumption of drugs Consumption of alcohol	Temporal removal of homesickness Fewer social networks and societal assistance Substance misuse/addiction Reduced personal control
**Approach 5: particularities of job mobility**	
Decisive step to leave Consideration given to poaching approaches by other companies Steady search for a new role in a rival company Second job/private consulting	Punish current employer Integrate a more friendly organisation community Be involved in new challenges Access greater mental health support Keep mind occupied

### Coping strategies

The participants reacted differently to evolving homesickness symptoms and developed their own coping mechanisms centred on five key aspects: socialisation, education, personal effort, medicalisation and job mobility—based on the data. These aspects are divided into two main categories: positive practices and (see Tables 3 and 4) and explained hereunder (Research Question 3). The positive and negative practices categories were arrived at by identifying in the main themes, aspects that enhanced the expatriates’ organisational and social integration (positive practices) and aspects that caused further isolation, stress and withdrawal (negative practices).

#### Positive practices

Positive practices (Table 3) capture homesickness coping approaches that have some positive impact on expatriate well-being.

#### Socialisation

The expatriates use social networks to deal with homesickness. Three participants interacted with various people, e.g. host country nationals and foreign colleagues from various countries to achieve a relatively psychologically balanced life. More participants admitted that having contacts with people that they were not familiar with did not yield the helpfulness they expected soon after locating. This is because they were searching for private spaces to help them organise their thoughts and find meaning in the current reality. These expatriates used counter-productive self-inflicted isolation.

Accompanied expatriates experienced less the symptoms of homesickness. Twelve respondents that kept regular contacts with close friends and family back home missed home less intensely than their counterparts who did not. Socialisation aspect is summarised in Table 3.

#### Education

This aspect encapsulates harmonised employer support, e.g. pre-departure preparation and briefings, pre-departure visits to host locations and some conversation with returnee expatriates. Many expatriates accepted that preparation afforded valuable insights of the country of assignment and the scopes of the new roles. Ten expatriates accepted that hearing from former assignees provided a good picture of the new place and reassurance. Four first-time-expatriates had overlaps with previous post-holders within the preparation for the posting, which allowed them to receive useful guidance from the sitting expatriates.

Cross-cultural management was an important part of the expatriate pre-assignment preparation. Expatriates who had such preparation, even limited in scope, believed that it had merits. Expatriates who had too little or no training felt left out and isolated in London. The expatriates who took formal cultural awareness courses had greater chances of meeting locals and live more socially and professionally fulfilling lives. The education strategy is shown in Table 3.

#### Personal effort

Personal effort has three main aspects: disposition to sustain host and home contacts, self-initiated informal cultural acquisition and utilisation of past experiences. The first aspect is about the participant attempting every possibility to remain in contact with newly formed relationships and home networks, using letters, email, phone, Skype and home visits. Another aspect of the personal effort approach (Table 3) is self-initiated cultural acquisition. Unconsciously or consciously, the participants constructed a knowledge acquisition programme intended to abate homesickness. The expatriates keenly invested in understanding the host societal and organisational practices. Some participants ‘got hooked up’ on host television programmes to learn British culture.

#### Negative practices

Negative practices (Table 4) capture homesickness coping approaches that do not relieve the expatriate from homesickness but on the contrary worsen their condition.

#### Medicalisation

Medicalisation was the least used strategy—only two of the participants used it. The expatriates who used this approach took medicines or drugs for most pains, even if when medication was not required. They took pills daily to treat fatigue, headaches and stomach-aches. A participant admitted taking pills to relax after a long day. The other expatriate took sleeping pills to ‘bury’ their obsessive thoughts about home. Table 4 summarises the medical approach.

#### Job mobility

The final aspect identified in the expatriates’ coping strategy is job mobility. This involved looking for a new job, sometimes with rivals. With the significant numbers of multinationals in London, expatriates can easily be poached by rival companies.

The expatriates’ coping strategies could reduce the negative impacts of homesickness on the expatriate and the organisation [47]. In the context of international HRM, Deresky [10] and Florkowski and Fogel [17] noted the need for adequate expatriate pre-departure preparations which should be complemented with practices and strong organisational HRM policy frameworks. HRM could embed such activities in a bundle of mechanisms to reduce the anxiety resulting from acculturative stress. Preparations could focus on information about host country realities and the possible temporary or protracted periods of psycho-social isolation. In attempting to reduce the occurrence of homesickness, expatriate briefings need to provide for regular home visits and building sociocultural networks in the expatriates’ new ‘home’ [34]. Thurber and Walton [47] advocate the maintenance of contacts with home, stressing family factors in preventing and treating homesickness.

#### Role of HRM in integrating Global South expatriates

Within the preparation initiatives, some have suggested the use of new technology for expatriates to keep in touch with family, friends and colleagues back home [35]—Research Question 2. However, other international HRM authors have argued that most expatriate literature continues to blithely ignore Skype, Facetime, WhatsApp, etc. [5]. Though new technologies are significant in mending some psychological fissures left by displacement, in the case of Global South expatriates, it appears that the cultural determinant is greater. But Dekker and Engbersen [9] do not perceive social media a fetish remedy for homesickness as it can have a double effect: both reduce homesickness or increase it. Thus, social media use could only be effective in combination with other positive practices; otherwise, if addiction to social media develops, it could become negative practice to soothe homesickness [32].

For Global South expatriates, reducing the negative effects of homesickness could involve the development of HRM frameworks encompassing headquarter and subsidiary cultural networks [41]. Many participants alluded to missing cultural aspects of the home country and family. This could be a sharp departure from the experience of traditional Western expatriates who are from individualistic countries and therefore could tolerate reduced extended family presence better than Global South expatriates whose cultures largely fall with collectivism. Some expatriates also pointed to racial prejudice which contributes to lower their confidence in operating in the host Western countries [6, 23]. This contributed to lower their status as experts and expatriates compared with Western expatriates who usually enjoy a higher status in emerging economies subsidiaries [44]. Women also suggested that more ethnic prejudice led them to feel more homesick, raising the possibility of a linkage between gender and homesickness in expatriation—an area that should command more research. Cultural networks help members to learn from their respective adjustment strategies [35]. Such successes, which can reduce the deleterious impacts of homesickness, ought to be captured and widely shared amongst current and future expatriates from different countries. In this perspective, the central role of the international human resource department is central [5]. There is a case for specifically adopting a systematic HRM approach to managing Global South expatriates. In fact, the medicalisation model found here is a key aspect affecting Global South expatriates, who due to cultural reasons, are reluctant to seek counselling or professional mental health assistance [1, 33].

For Global South expatriates specifically, cultural spheres, e.g. social clubs, religious and educational institutions can have a positive effect on the mental health, performance and commitment of the expatriates if these sociocultural resources are effectively researched and deployed by HRM to create dynamic capabilities because HRM is a driver for fulfilling organisational strategy [52]. Sociocultural resources publicised by HRM could be important ‘healing’ networks for dislocated and homesick expatriates.

## Research implications and limitations

The research has practical implications in that it can help international HRM within organisations to formulate policies and development strategies that contribute to better mental health for expatriates, thus seeding the ingredients for greater performance and job satisfaction. Within the preparation initiatives, the use of new technology can be critical to keep in touch with family, friends and colleagues back home. This study’s results reject the linearity in the homesickness model presented by Fisher [15] and detailed in our theoretical framework. Authors such as Stroebe et al. [45], Van Vliet [55] and Duven [12] also reject the linear perspective in homesickness. The expatriates developed homesickness at different stages of the expatriate assignment and the symptoms varied between participants. Critically, some have gone through a process of healing using some positive practices identified in our model but they subsequently relapsed and returned to the initial stage of their condition. These fluctuations command that homesickness be treated as a revolving issue and the unpredictability of its occurrence at any stage of the expatriate assignment means that continuous support packages remain in place.

The research’s main limitation centres on the small sample used. Though this limitation was mitigated by the diversity of sectors and nationalities of the participants, wider studies, perhaps drawing on quantitative studies using large sample sizes will allow for more generalisation. Such studies will be strengthened by a cross boarder approach, looking at participants in various Western developed countries. With increasing talent being sourced in the developing world due to labour shortages in the West, at the same time as expanding international business to culturally and geographically distant localities, HRM is key to [organisational] mission accomplishment [52]. Academic and HRM practitioner research are axiomatic to better understand the adaptation issues of Global South expatriates given the recentness of increased deployment of developing world expatriates to the West.

## Conclusion

The research answers the first research question by demonstrating that homesickness is a key issue and prevalent in expatriates from the Global South operating in advanced economies such as the UK. Homesickness has significant negative personal and organisational ramifications when there are no systematic HRM interventions, and institutional framework to support expatriates with different needs, i.e. Global South expatriates. This is linked to the insufficiency of expatriate preparation prior to departure, the deficiency of in-assignment support generally [5] and culture distance.

In the absence of systematic organisational support mechanisms, Global South expatriates are left to devise individual strategies for coping with homesickness. When this happens, uncoordinated untested individual strategies could, in some cases, be detrimental to the individual performance. For instance, in this study, medicalisation has proved to worsen the homesickness condition. Even the other approaches that showed a degree of success, presented some issues, e.g. state of dependency and disconnection with work since more effort is deployed trying to combat homesickness as opposed to focusing on the expatiate role. More significant at the individual level is the impact on the individuals’ health and well-being. Expatriation is designed to enhance careers, creating a sense of achievement [26]. If the psychological effects of expatriations are inadequately managed by HRM, the knock-on effect is the long-term damage of the expatriates’ professional confidence, family and social networks. HRM has a duty to ensure the expatriates’ new location has a sense of ‘home’.

Homesickness can pause challenges for both Global South expatriates and their organisations. These challenges include decrease team dynamic and performance, loss of contacts and networks and decrease organisational commitment. Further, failed expatriation can deter potential good expatriates, thus reducing the talent pool [10, 26].

## Data Availability

Please contact author for data requests.
